# A New Adjuvant Treatment for Glioblastoma Using Aprepitant, Vortioxetine, Roflumilast and Olanzapine: The AVRO Regimen

**DOI:** 10.3390/ijms26136158

**Published:** 2025-06-26

**Authors:** Richard E. Kast, Bruno Marques Vieira, Erasmo Barros da Silva

**Affiliations:** 1 IIAIGC Study Center, Burlington, VT 05408, USA; 2 Brain Biochemistry Laboratory, Instituto Estadual do Cérebro Paulo Niemeyer, Rio de Janeiro 20231-092, Brazil; brunomarquesv@gmail.com; 3 Neurosurgery Department-Neuro-Oncology, Instituto de Neurologia de Curitiba, Rua Jeremias Maciel Perretto, 300-Campo Comprido, Curitiba 81210-310, Brazil; erasmo-inc@uol.com.br

**Keywords:** cAMP, dopamine, glioblastoma, multidrug, NK-1, repurposed drugs

## Abstract

AVRO is an adjunctive four-drug regimen designed to increase the effectiveness of current standard treatment of glioblastoma (GB). AVRO is a repurposed drug regimen consisting of the antinausea drug aprepitant, the antidepressant vortioxetine, the emphysema treatment drug roflumilast, and the antipsychotic drug olanzapine. All four are EMA/FDA approved for nononcology indications, all four have strong research evidence showing inhibition of GB growth, and all four carry a low side effect risk. The goal of adding AVRO is to further retard GB growth, improving survival. Aprepitant is an antinausea drug that blocks NK-1 signaling, with a database of 59 studies showing growth inhibition in 22 different cancers, 12 of which were specific to GB. Fully 30 studies demonstrated that the SSRI class of antidepressants inhibited GB growth; accordingly, we chose one such agent, vortioxetine, to add to AVRO. Elevation of intracellular cAMP slowed GB growth in 21 independent studies. Accordingly, we added the emphysema treatment drug roflumilast, which inhibits cAMP degradation. Among the 27 currently marketed D2-blocking antipsychotic drugs, 24 have preclinical evidence of GB growth inhibition in a combined 84 independent study database. One of these 24 drugs is olanzapine, added to AVRO. Given the short median survival of GB as of mid-2025, the clinician and researcher community will benefit from wider awareness of the anti-GB effects of these four nononcology drugs.

## 1. Introduction

The marked intrinsic invasiveness and the extreme molecular heterogeneity of glioblastoma (GB) limit the effectiveness of current treatments. Microscopic islands of GB cells are found throughout the entire brain at the time of diagnosis. With the current standard of care, consisting of macroscopic tumor resection, irradiation, temozolomide, and the use of the Optune™ device, which delivers low-energy radio frequency nonionizing irradiation, median GB survival remains less than two or three years after diagnosis [1,2,3]. A remarkably large array of clinical trials of different chemotherapies, immunotherapies, irradiation schedules, and kinase inhibitors and combinations of these regimens have failed to improve this dismal survival rate.

AVRO is an adjunctive four-drug regimen designed to increase the effectiveness of the current standard of care. It consists of the antinausea drug aprepitant, the antidepressant drug vortioxetine, the antipsychotic drug olanzapine, and the emphysema treatment drug roflumilast. At first glance, it may seem strange that these four nononcology drugs interfere with GB growth, but as we outline here, the database does show that they do so. The biochemical/physiological rationale behind each drug’s GB inhibition is sound.

As a contribution to the school of thought in modern oncology that a multidrug chemotherapy regimen will be needed to effectively halt growth in the common currently incurable cancers, we present in this paper the AVRO regimen. AVRO augments standard treatment by targeting diverse yet complementary aspects of GB biology, including inflammation, neurotransmission, and oncogenic signaling, through the repurposing of clinically approved drugs. AVRO has a favorable safety profile and, as we show here, a physiologically based rationale.

The reason why multiagent chemotherapy is needed to address the different subpopulations within a GB and the growth drive flexibility of GBs, has been reviewed by us and others [4,5,6,7,8,9]. AVRO represents our further thoughts on GB physiology, with additional data on drugs and deeper insights into GB pathophysiology since the discovery of CUSP9v3 [10]. Four-drug AVRO is less daunting than the ten-drug regimen CUSP9v3. AVRO is an alternative regimen, not a replacement of CUSP9v3.

## 2. Aprepitant

NK-1 is a ubiquitous 11 amino acid signaling molecule that is synonymous with Substance P. Aprepitant is an NK-1 receptor inhibitory drug that is marketed and commonly used for the reduction of nausea and vomiting during emetogenic chemotherapies [11,12,13,14,15]. NK-1 strongly colocalizes with serotonin in gut enterochromaffin cells but is widely expressed throughout the body.

Emetogenic chemotherapies increase the blood levels of NK-1 [16,17]. Given that, as we show below, NK-1 is a growth driver in GB, controlling nausea becomes important and a valuable addition throughout the course of GB. Aprepitant has a long half-life (days) and few side effects. Aprepitant is metabolized primarily by CYP3A4 but also inhibits CYP3A4, contributing to its long half-life [18,19]. A plasma aprepitant concentration of 10 ng/mL resulted in 50% brain NK-1 receptor occupancy; 100 ng/mL resulted in 90% occupancy [13]. These plasma levels are readily obtained in aprepitant’s clinical use. GB tumor tissue NK-1 receptor occupancy after aprepitant has not yet been studied.

We added aprepitant to the AVRO regimen on the basis of the strong dataset showing that aprepitant inhibits NK-1 signaling and that NK-1 signaling forms part of the suite of growth-driving forces in GB. Three currently marketed drugs inhibit NK-1 signaling at its receptor: aprepitant, fosaprepitant, and rolaprepitant. We present here only data collected specifically on aprepitant. The role of NK-1, and its signaling at the NK-1 receptor as part of a suite of signaling systems driving GB growth, was recognized three decades ago. Data on this topic have been reviewed periodically since then [20,21,22,23,24].

NK-1 signaling at its receptor promotes or facilitates malignant growth and metastasis as a general phenomenon observed in common human cancers, as shown in Table 1. In light of this and the good tolerability of aprepitant, the risk/benefit balance favors adding aprepitant to the current GB standard of care as part of a multidrug adjunctive regimen. Table 1 shows references to 59 published studies of growth inhibition by aprepitant in 22 different cancers, indicating that NK-1 may be a pan-malignant growth element. Overall, 12 of these 59 studies demonstrated growth inhibition specifically in GB [9,25,26,27,28,29,30,31,32,33,34,35]. After reviewing several of these studies, Munoz and Russo concluded in 2025 that “…all glioma cells express NK-1R, and NK-1R is essential for the viability of glioma cells and not of normal cells” [24]. Typically, in these studies, the GB growth inhibition IC50 of aprepitant lies in the range of 10–40 μM.

As a corollary to the data in Table 1, a study reported that the addition of exogenous NK-1 to cell cultures stimulated growth and enhanced in vitro migration in cells of acute lymphocytic leukemia, breast cancer, cervical cancer, colon cancer, gastric cancer, GB, laryngeal cancer, melanoma, ovarian cancer, neuroblastoma, small cell and non-small cell lung cancer, pancreatic cancer, and prostate cancer [28,30,36,37,38,39,40,41,42,43,44,45,46,47,48,49].

**Table 1:** References to aprepitant growth inhibition across 22 different cancers. ALL, acute lymphoblastic leukemia; AML, acute myelogenous leukemia; APL, acute promyelocytic leukemia; cholangio, cholangiocarcinoma; CML, chronic myelogenous leukemia; NSCLC, non-small cell lung cancer; SCLC, small cell lung cancer.

Cancer Type	Aprepitant Effects, References
ALL	apoptosis, cytostatic, additive with doxorubicin [49,50]
AML	inhibited in vitro and xenograft growth [51,52,53,54]
APL	growth inhibition and additive with vincristine [55,56]
breast	inhibited in vitro and xenograft growth [30,36,57,58,59,60,61,62,63]
cervical	inhibited growth in vitro [37,64]
cholangio	inhibited growth, in vitro and xenograft [65,66]
CML	apoptosis and decreased colony formation [52,54]
colon	inhibited exosome release, xenograft growth [59,67,68,69,70]
esophageal	inhibited growth in vitro and xenograft [71,72]
glioblastoma	xenograft and potential clinical growth inhibition [10,25,26,27,28,30,31,32,33,34,35,73]
hepatoblastoma	stem cell inhibition by Wnt suppression [74,75]
hepatocellular	in vitro cytotoxicity and xenograft inhibition [76]
melanoma	in vitro growth inhibition [77]
myeloma	inhibited metabolism and growth in vitro [56,78]
neuroblastoma	xenograft growth inhibition [47,79]
NSCLC	NK-1 stimulated aprepitant inhibited growth in vitro [80,81]
osteosarcoma	inhibited growth in vitro [82,83]
ovarian	inhibited growth, synergy with doxorubicin in vitro [39,40]
rhabdoid	growth inhibition and apoptosis in vitro [84]
pancreatic	inhibited growth and motilityin vitro [85,86]
prostate	inhibited growth in vitro [87,88,89,90,91]
SCLC	NK-1 stimulated aprepitant inhibited growth in vitro [41]

It should be appreciated that there is no other nontoxic molecule or marketed drug that has so little effect on normal cells yet inhibits growth of such a wide variety of human cancers. Aprepitant is unique.

Both NK-1 overexpression and NK-1 receptor overexpression are common findings across human cancers [73,92,93,94,95]. All patients with GB heavily expressed NK-1 receptors according to immunohistochemical biopsy analysis [96]. Breast cancer patients with low NK-1R expression according to immunohistochemical analysis of biopsy tissue survived longer than those with high NK-1R expression [97].

On the basis of an extensive database showing that NK-1 signaling contributes to malignant growth across several different cancer types, the repurposing of NK-1 signaling to inhibit cancer growth was straightforward [98,99,100].

The additional benefit of adding aprepitant to any GB treatment is its ability to reduce brain edema. Excess NK-1 release drives much of the brain edema after traumatic brain injury, and accordingly, an experimental NK-1 inhibitor, n-acetyl-L-tryptophan, reduces that edema [101,102,103]. NK-1 overexpression contributes to the peritumoral edema of experimental melanoma brain metastases, and accordingly, aprepitant reduces that edema [104,105]. Similarly, an experimental NK-1 antagonist (EU-C-001, PresSura Pharmaceuticals) reduced experimentally induced ischemia-related intracranial pressure elevation and peri-infarct edema in sheep [106]. NK-1 antagonism as a treatment for brain edema was reviewed in 2013 [107]. Data on the potential MOA come from lungs exposed to hypoxia that begin overexpressing NK-1 concomitantly with increased cytokine release and edema formation [108]. This response to the hypoxic areas of the GB, which are likewise associated with edematous areas, could explain the reduction in edema in the GB caused by aprepitant. Why aprepitant is not currently used for brain edema reduction has never been explained in print. Olanzapine added to aprepitant regimens was safe and provided additional nausea control after cisplatin [109].

Perhaps the strongest evidence for the role of NK-1 in promoting cancer growth comes from six clinical studies of serum NK-1 levels in human cancers. The serum NK-1 concentration was 13 ± 3 ng/mL in papillary thyroid carcinoma patients and 6 ± 2 ng/mL in controls [110]. A second study reported that the serum NK-1 concentration was 14 ± 4 ng/mL in colon cancer patients and 5 ± 1 ng/mL in controls [111]. A third study reported 19 ± 5 ng/mL in colon cancer patients and 1 ± 0.2 ng/mL in controls [112]. A fourth study reported that the serum NK-1 concentration was 10 ± 3 ng/mL in endometrial cancer patients and 5 ± 2 ng/mL in controls [113]. A fifth study reported that the serum NK-1 concentration was 17 ± 12 ng/mL in bladder cancer patients and 2 ± 2 ng/mL in controls [114]. A sixth study reported that the serum NK-1 concentration was 16 ± 3 ng/mL in breast cancer patients and 5 ± 1 ng/mL in controls [115]. These dramatic results are notable on several accounts: (i) the close concordance between several studies, (ii) the similarity of findings across different cancers, and (iii) the unusual finding of nonoverlapping ranges between controls and patients.

ACE, DPP-IV, and neprilysin are three endopeptidases that degrade NK-1 [116,117,118,119,120]. Therefore, ACE inhibitors and DPP-IV inhibitors (“gliptins”) are best avoided during cancer treatment or during treatment in any setting where NK-1 has been shown to play a pathogenic role.

In three healthy humans, a single dose of oral aprepitant at 100 mg resulted in plasma levels of 0.08, 0.5, and 1.1 μg/mL, corresponding to brain NK-1 receptor occupancies of 91%, 95%, and 94%, respectively [121]. Coadministration of ritonavir with aprepitant 375 mg per day resulted in plasma aprepitant levels of 31 μg/mL on day 14 and 23 μg/mL on day 28, reflecting the induction of aprepitant metabolism [122]. Aprepitant 375 mg per day for 14 days without ritonavir resulted in plasma aprepitant levels of 8 μg/mL, reflecting the major effect of ritonavir’s inhibition of aprepitant metabolism [122].

The addition of 125 mg of continuous aprepitant once daily indefinitely during the entire course of GB treatment and follow-up has the potential to prolong survival, particularly if it is used as part of a multidrug regimen. It is predicted to be well tolerated if known drug-drug interactions are taken into consideration when other drugs are dosed. On the basis of the data in Table 1 and the assembled data here, NK-1 receptor inhibition with aprepitant may be active in inhibiting many different cancers.

## 3. Vortioxetine

Vortioxetine is an antidepressant related to the serotonin reuptake inhibitor group (SSRIs; citalopram, fluoxetine, fluvoxamine, paroxetine, and sertraline). Vortioxetine inhibits the reuptake of serotonin, as do the other SSRIs, but it has meaningful differences from the other SSRIs. The side effect profile of vortioxetine differed from that of the other drugs in the SSRI group. Although all of these drugs inhibit the serotonin reuptake pump on neurons and glia, vortioxetine has a lower incidence of sleep disturbances, sexual dysfunction, weight gain, and only rare discontinuation symptoms [122]. Vortioxetine has a half-life of 2–3 days. Vortioxetine also inhibits the serotonin receptors 5-HT1D, 5-HT3, and 5-HT7 and has partial agonist activity on the 5-HT1A and 5-HT1B receptors, an attribute lacking in other SSRIs [123,124,125]. Previous reviews of GB growth inhibition by pharmacologic inhibition of 5-HT7 receptors have been published [126,127,128]. Vortioxetine is also safe and effective when used as an antidepressant in people with cancer [129].

**Figure 1:**
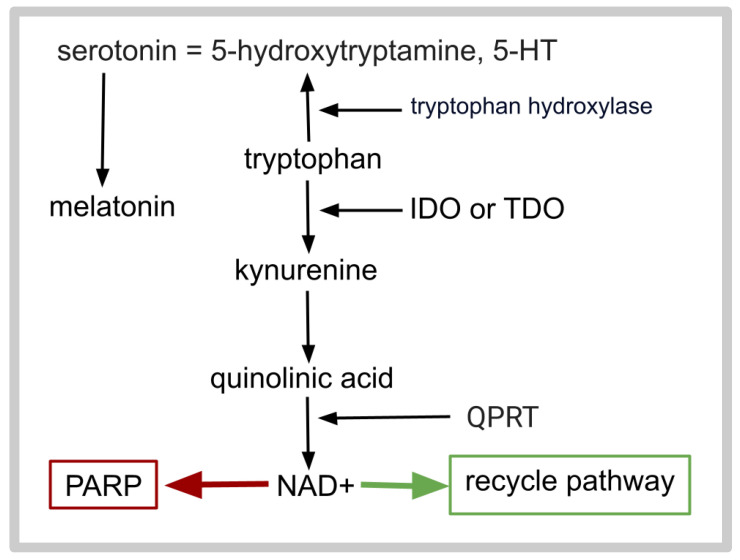
Basic outline of a metabolic branchpoint leading to either the kynurenine pathway or the serotonin–melatonin pathway. Many intermediates, mediating enzymes, and offshoots have been omitted from this schematic. IDO, indoleamine dioxygenase; TDO, tryptophan dioxygenase; PARP, poly ADP-ribose polymer. PARPs catalyze the reaction of ADP-ribose from nicotinamide adenine dinucleotide (NAD+) to targets; QPRT, quinolinic acid phosphoribosyltransferase.

Vortioxetine’s inhibition of GB growth could be an SSRI class phenomenon in that the related SSRIs fluoxetine and sertraline have extensive databases showing growth arrest in GB cells—for example, fluoxetine [130,131,132,133,134,135,136,137,138] and sertraline [9,28,30,34,35,139,140,141,142,143,144,145]. A study by Bielecka-Wajdman et al. revealed that 10 µM fluoxetine reduced several GB stem cell markers without reducing viability [146]. Figure 1 outlines several core elements of tryptophan metabolism beyond its use in proteins. The main branch point for tryptophan metabolism leads to either serotonin and melatonin synthesis or to the kynurenine pathway.

Vortioxetine inhibited gastric cancer cell growth by reducing the kinase activity of JAK2 and Src [147], but other data, while confirming growth inhibition, indicated that the inhibition of gastric cancer cell growth by vortioxetine involved PI3K/AKT [148].

In 2024, two studies addressed the effects of vortioxetine specifically in GB. A large chemical library screen identified vortioxetine as having the strongest synergy with temozolomide in GB inhibition [149]. Another in vitro study showed that growth, migration and cell survival were suppressed by 5 to 12 µM vortioxetine alone [150]. A 2025 study identified vortioxetine as having an in vitro IC50 of between 5 and 12 µM, depending on the GB cell line tested [151]. They identified PI3K/AKT inhibition as the MOA, in accordance with the gastric cancer studies referenced above. A detailed pathway analysis by Zhuo et al. in 2025 outlined the intersection of the targets of vortioxetine and the GB growth networks inhibited by vortioxetine [152]. A 2025 study by Wang et al. revealed some in vitro growth inhibition of nontransformed normal astrocytes (IC50 of 4.5 µM), but for human GB cells, the cytotoxicity of vortioxetine was half that (IC50 of 1.9 µM) [153]. Notably, we have no evidence of vortioxetine toxicity to normal brain cells during clinical use in tens of thousands of patients.

Vortioxetine decreases quinolinic acid production, but the locus and MOA of this effect is unknown [154]. Lowering quinolinic acid levels is a worthwhile goal during GB treatment for several reasons. Quinolinic acid, an excitatory N-methyl-D-aspartate (NMDA) glutaminergic receptor agonist, is neurotoxic [155,156,157,158,159]. Shifts toward the kynurenine pathway increase GB’s immunosuppressive indoleamine 2,3-dioxygenase (IDO) synthesis [158]. Quinolinic acid phosphoribosyltransferase (QPRT) is the rate-determining enzyme for the de novo synthesis of NAD+ from tryptophan via the intermediate quinolinic acid, an upregulated system in GB that furthers GB cell survival [159]. The resistance of GB cells to histone deacetylase inhibitors such as panobinostat depends on fully functioning quinolinic acid for NAD+ biosynthesis [160]. Quinolinic acid-induced NMDA glutamate receptor activation in macrophages triggers a more supportive tumor phenotype [161].

## 4. Roflumilast and PDE4

**Figure 2:**
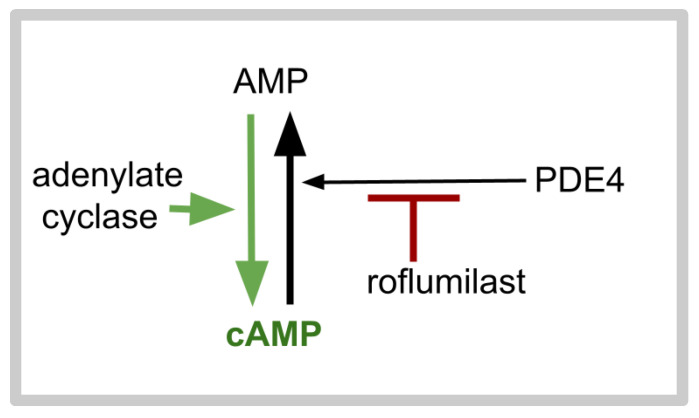
Basic outline of the action of cAMP and roflumilast. The phytochemical forskolin, or agonism at dopaminergic D1 or D5 receptors increase the activity of adenylate cyclase. D2 dopaminergic receptor agonism decreases adenylate cyclase activity. Apremilast, rolipram, and roflumilast inhibit the conversion of cAMP to AMP mediated by phosphodiesterase 4 (PDE4), represented by the black up-pointing arrow.

Roflumilast, introduced in clinical practice two decades ago, is a phosphodiesterase 4 (PDE4) inhibitor that is well tolerated and approved for treating psoriasis and emphysema [162,163,164,165]. In a dozen randomized clinical trials, side effects did not significantly differ from those of placebo [166]. PDE4 has several isoforms, each of which is a product of different genes [167,168]. By inhibiting PDE4, roflumilast increases the level of intracellular cAMP by diminishing the conversion of cAMP to AMP, as depicted in Figure 2.

Adenylate cyclase catalyzes the conversion of AMP to cAMP. PDE4 catalyzes the reverse reaction, the conversion of cAMP to AMP. Agonism at D5 increases cAMP synthesis by activating adenylate cyclase. Agonism at D2 decreases cAMP synthesis by deactivating adenylate cyclase. In light of Prabhu et al.’s demonstration that a greater D5 to D2 ratio is associated with longer survival in GB, we conclude that lowering intracellular cAMP enhances GB growth and that increasing intracellular cAMP slows GB growth and that the choice of antipsychotic drug should be guided by this; i.e., it is best to use a drug heavily weighted to D2 rather than D1 blockade. A paper in 2011 reviewed evidence of an inverse relationship between a glioma’s intracellular cAMP level and its malignancy grade, suggesting that increasing cAMP levels via PDE4 inhibition is a treatment approach for GB [169]. We add here data collected since then.

In addition to roflumilast, we have clinical experience with two other PDE4 inhibitors, rolipram and apremilast. Rolipram has been clinically tested as an antidepressant, for which it was effective, but rolipram has never been marketed. Apremilast is a PDE4 inhibitor marketed and used for indications (psoriasis and emphysema) similar to those for roflumilast. The same rationale as for roflumilast would apply to apremilast or rolipram as an adjunct to GB treatment. This was reviewed in 2018 [170].

Fully ten independent studies have shown that the PDE4 inhibitor rolipram inhibits in vitro or murine graft GB growth [171,172,173,174,175,176,177,178,179,180]. Two of these studies were in vivo murine graft studies demonstrating GB growth inhibition; Dixit et al. reported that rolipram inhibited GB xenograft growth but not in vitro growth [171], and Goldhoff et al. reported less orthotopic xenograft GB growth with rolipram, temozolomide, and irradiation than in controls receiving only temozolomide and irradiation [173].

Forskolin is a phytochemical that forms hydrogen bonds with adenylate cyclase, stabilizing the catalytic area of adenylate cyclase in the active conformation and protecting it from inactivation, thereby increasing the level of intracellular cAMP [181,182,183].

Ionizing radiation induces cancer stem cell attributes in some of the surviving nonstem cancer cells [184,185,186,187,188]. He et al. reviewed data specifically on GB cell irradiation increasing stem cell attributes in some surviving cells [188]. Forskolin’s increase in cAMP redirected this irradiation-induced plasticity away from stemness toward differentiation into microglia and neuronal phenotypes [188]. Survival after GB grafting increased, and the GB stem cell population decreased in the grafted mice receiving irradiation + forskolin compared with those in the grafted and irradiated groups. Data from the 1980s demonstrated that increasing intracellular cAMP promoted GB cell differentiation toward a neuronal phenotype [175,189]. Forskolin alone decreased viability and was an additive with temozolomide in some, but not all, GB cell lines [190].

Dibutyryl cAMP has a cAMP-related end effect that is similar to that of roflumilast, apremilast, and rolipram. Dibutyryl cAMP is a cell-permeable cAMP mimic that resists PDE4 degradation. It triggers a decrease in GB cell aerobic glycolysis and an increase in the number of mitochondria and promotes GB differentiation into neuron-like cells [191,192]. Forskolin also decreases GB cell aerobic glycolysis, increases the number of mitochondria, and prompts their differentiation into neuron-like cells [192]. Similarly, increased intracellular cAMP by either adenylate cyclase activation by forskolin or the addition of dibutyryl cyclic AMP decreased GB cell migration and the in vitro growth rate of GB cells [193]. However, while confirming that dibutyryl cAMP triggered senescence markers and differentiation toward neuron-like cells in vitro, others reported that dibutyryl cAMP increased the extracellular acidification rate and IL-6 secretion [194].

The role of cAMP signaling in cancer generally cannot be stated categorically. Both growth promotion and growth suppression in response to increased intracellular cAMP are recognized, depending on the experimental conditions, cancer type, and stage [195]. Perhaps most importantly, as with most of our other interventions, changing cAMP signaling can simultaneously engage both growth-inhibiting and growth-stimulating elements. It is the net effect that determines our decisions to use, and when to use an intervention or not: cf. the chess aphorism “all moves create strengths and weaknesses”.

## 5. Olanzapine, D2 Dopamine Receptors, cAMP, and GB

Multiple convergent lines of evidence indicate that dopamine receptor D2 agonism increases GB growth. Here, we report that at least one of the MOAs that enhance GB growth is D2. Among the 27 antipsychotic drugs marketed and used worldwide to block or reduce D2 signaling, 24 have preclinical data showing GB growth inhibition, as listed in Table 2. Empirically, 84 experimental studies of these 24 different currently marketed D2 blocking antipsychotic drugs have shown that GB growth is inhibited by the respective studied drugs. Convergent lines of evidence that blocking D2 with antipsychotic drugs increases the level of intracellular cAMP, thereby interfering with GB growth, are discussed below.

There are five subtypes of receptors activated by dopamine: D1, D2, D3, D4, and D5. These receptors are divided into two groups: the D1 and D5 group, which couple to Gαs, increasing adenylate cyclase activity, and the D2, D3, and D4 group, which couple to Gαi/o, decreasing adenylate cyclase activity [196,197].

There are, however, other potential second messengers from these dopamine receptors. D2 includes a second messenger branch point leading to either Gi/o or β-arrestin [196,198]. Six independent studies have reported D2 receptor overexpression in GB tissue compared with that in the surrounding brain [199,200,201,202,203,204]. GB cells themselves synthesize dopamine [200,205]; thus, we have evidence that autocrine dopaminergic signaling contributes to GB growth drive.

Table 2 lists 84 studies of clinically used antipsychotic drugs that show experimental GB growth inhibition. All inhibit D2 signaling. These studies attributed GB inhibition to several MOAs, but the only common denominator among all 24 drugs in all 84 studies was D2 blocking. Among these 24 antipsychotic drugs, we chose olanzapine for AVRO on the basis of (i) its good tolerability and (ii) its ability to increase appetite, inhibit nausea, and improve sleep quality.

**Table 2:** List of 27 FDA- and/or EMA-approved D2 blocking drugs that are used to treat psychosis. Of these 27 currently marketed D2 blocking antipsychotic drugs, 24 have preclinical evidence of GB growth inhibition in a combined 84-study database, the references of which are listed below. The three antipsychotic drugs that do not inhibit GB growth have not been tested for such activity.

Drug	2020 to 2024	Prior to 2020
aripiprazole	---	[206,207]
asenapine	[208]	---
brexpiprazole	---	[209,210]
cariprazine	---	---
chlorpromazine	[141,211,212,213,214,215]	[216,217,218,219,220,221]
clozapine	---	[222]
droperidol	---	---
fluphenazine	[141]	[219,220,223]
fluspirilene	[129,223,224]	[225]
haloperidol	[226,227,228,229,230,231]	[231,232]
iloperidone	[233]	---
levomepromazine	[230]	---
loxapine	---	[219]
lurasidone	[234]	---
metoclopramide	---	[235,236,237]
molindone	---	[219]
olanzapine	[238]	[239,240,241]
paliperidone	[242]	---
penfluridol	[129]	[220,243,244,245]
perphenazine	[246,247]	[130,248,249,250]
pimozide	[129,203,229,251,252,253,254,255]	[256,257,258,259,260]
prochlorperazine	[247]	[248]
quetiapine	[34,261]	[262,263]
risperidone	[242,264,265]	[227,266]
thioridazine	[229,267]	[268,269,270,271]
thiothixene	---	---
trifluoperazine	[272,273,274,275]	[216,276,277]

Crucially, for GB treatment, temozolomide exposure increases GB cell D2 expression [204,205,226,227]. This was associated with a decrease in intracellular cAMP, as expected from increased D2 signaling via Gi/o inhibition of adenylate cyclase. The D2 blocking antipsychotic drug haloperidol reversed the decrease in cAMP and was synergistic with temozolomide in mediating GB cell cytotoxicity [226]. These results reflect a core aspect of mammalian homeostatic physiology: a disturbance of a given state tends to provoke compensatory changes to restore the state prior to the disturbance.

D2 readily heterodimerizes [278,279]. Although the heterodimer partners for D2 specifically in GB have not yet been determined, in other contexts, some of the heterodimer partners previously identified are adenosine A2A [198], CB1 cannabinoid [280], oxytocin [281], bradykinin receptor B2 [282], 5HT2 [198], and importantly, for GB pathophysiology, heterodimers with dopamine receptor D5 [283]. D2-D5 heterodimers are particularly important for understanding GBs because agonism at D5 opposes or reduces the effects of agonism at D2. Prabhu et al. provided a demonstration of this phenomenon. They showed that ONC201, a D2-inhibiting drug developed for GB treatment, was more effective in those with higher D5-D2 dimer expression [283].

We now understand many elements of the physiological, electrochemical brain changes of psychosis that mediate disordered cognition, perception, and affect. Specifically, inhibiting brain D2 signaling commonly stops or reduces overtly psychotic elements [284,285]. We have many D2 blocking drugs due to the ~1% prevalence of psychosis across human societies worldwide.

**Figure 3:**
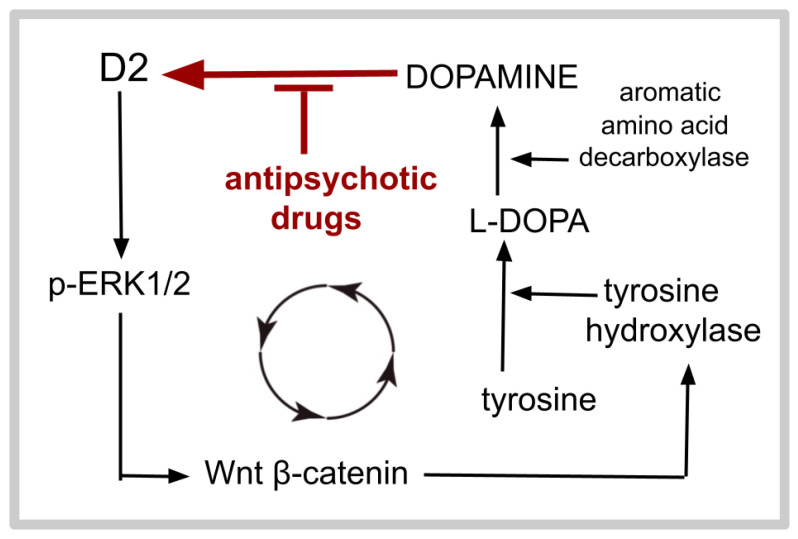
Schematic diagram of the feedback loop operating in GB, as reported by Yan Wang et al. [203]. Several intermediate steps, offshoots, and cofactors are omitted from the diagram.

A recently recognized D2-centered amplifying feedback loop operates in GB, as shown in Figure 3. This GB signaling loop was recently reported by Yan Wang et al., who demonstrated that dopaminergic signaling at D2 activates the signaling hub ERK, which results in the nuclear upregulation of tyrosine hydroxylase, a required enzyme that catalyzes the conversion of L-DOPA to dopamine [286]. The resulting increase in dopamine synthesis in turn increases signaling at D2. ERK overdrive is a core aspect of GB pathophysiology [126,246,287].

The prediction in 2020 that the antipsychotic drug perphenazine would augment temozolomide-mediated growth inhibition of GB was recently experimentally confirmed [246,288,289]. The primary evidence supporting the addition of a D2 blocking antipsychotic drug to GB treatment might be the empirical demonstration in the 84 research papers in Table 2, in which 24 different drugs that inhibit GB growth have one thing in common: they block D2 receptors.

The ideal antipsychotic drug in Table 2 for use during GB treatment has not yet been determined. We suggest olanzapine as a good drug to start clinical testing on the basis of its ancillary potential benefits and good tolerability in nonpsychotic patients.

Olanzapine is an inexpensive, generically available antipsychotic drug used widely to treat schizophrenia and other psychoses around the world. At a blood level of ~20 ng/mL 12 h post-dose, olanzapine occupies 65–80% of brain D2 receptors [290]. Olanzapine is an inverse agonist at D2; i.e., it reduces baseline and unliganded D2 activity and correspondingly increases intracellular cAMP [291]. Olanzapine’s inhibition of D2 exceeds its inhibition of D1 [292,293]. The brain tissue levels of olanzapine are 2 to 15 times greater than the plasma levels [294,295].

Olanzapine is usually well tolerated in psychiatric and nonpsychiatric use. Olanzapine is a potent antinausea drug and is frequently used as an effective addition to other antinausea medicines during emetogenic chemotherapies [296,297,298]. Olanzapine also reduces nausea during temozolomide treatment of GB.

Studies have shown that olanzapine provides better control of nausea than does aprepitant during highly emetogenic chemotherapy for myeloma [299]. Olanzapine combined with aprepitant provided better nausea suppression during highly emetogenic chemotherapies than did aprepitant alone [300]. In cases of intractable nausea, the addition of olanzapine to those with a partial response to triple therapy with aprepitant, ondansetron, and dexamethasone converts them to full responders [301].

Almost all psychiatric patients and many oncology patients will gain weight after starting olanzapine due to olanzapine’s strong appetite stimulation, which is an unwanted side effect of its psychiatric use but an effect that could become advantageous during GB or other cancer treatments. Unwanted side effects of extrapyramidal movement disorders and affective blunting can occur but are not common with olanzapine [302].

In cancer-related anorexia unrelated to pain or to chemotherapy, the addition of adjunctive olanzapine frequently improves appetite and quality of life without increasing the burden of side effects [296,303,304,305]. Further benefits of olanzapine not seen with most other D2 blocking drugs are (i) increases in sleep efficiency, (ii) increased duration of slow wave sleep, (iii) increased duration of total time asleep, and (iv) increased enjoyment of eating [304,306]. Notably, poor or inadequate sleep is common in GB patients and tends to cluster with fatigue, depression, cognitive impairment, and shortened survival [307,308,309].

Summary of data on D2, D5, and cAMP in GB:

- Agonism at D5 increases, agonism at D2 decreases cAMP production. A greater D5 to D2 ratio is associated with longer survival in GB.
- D2 agonism decreases intracellular cAMP and enhances GB growth.
- D2-blocking drugs such as olanzapine diminish the D2 mediated inhibition of adenylate cyclase. This is the primary theory on how antipsychotic drugs decrease GB growth.
- Several different drugs (rolipram, forskolin, and dibutyryl cAMP) that increase the intracellular cAMP level via different MOAs slow GB growth both in vitro and in rodent graft studies.
- Rolipram, apremilast, and roflumilast are related PDE4 inhibitors. Ten studies demonstrated the ability of rolipram to inhibit GB growth.

Several different MOAs for D2 inhibition-mediated GB growth inhibition have been shown in in vitro studies. No consensus exists on which effects are primary effects and which are secondary phenomena. Although possible, we believe that these GB growth inhibition MOAs are unlikely to be independent direct effects of D2 inhibition. More likely, they are downstream effects of a limited number of primary D2 receptor signaling losses. The 24 drugs listed in Table 2 that have data on GB inhibition belong to different chemical classes, have different side effect profiles, and have different receptor binding profiles to other neuronal or glial receptors. D2 inhibition is the only common denominator among these drugs.

An interesting further aspect favoring the use of D2 blocking drugs in GB is the effect of these medicines on eosinophils. In psychosis treatment, for several months after starting olanzapine or the archetypal antipsychotic drug haloperidol, patients’ eosinophil counts gradually increase [310]. Both the absolute eosinophil count and the eosinophil count as a percentage of total leukocytes slightly increase weekly, reaching a plateau at or near the upper range of normal. This condition is almost always asymptomatic, although isolated case reports of eosinophilic pleural effusions do exist. In a 2020 study, Vaios et al. reported that in newly diagnosed GB patients receiving standard treatment followed by bevacizumab, those with higher eosinophil counts survived longer than those with lower eosinophil counts [311]. The effect was not trivial. Multiple studies have confirmed the association of a higher absolute eosinophil count with longer GB survival [312,313,314,315].

## 6. Discussion

Clinicians decide to adopt a treatment on the basis of the preponderance of evidence and the consideration of balancing risks of treatment versus risks of the target disease. This consideration reflects an old wisdom expressed in the myth of Scylla and Charybdis. As a metaphor, we face these two monsters today when confronting GB or any metastatic cancer. The Ionian poet Homer (~800 BCE) advises that we pass by Scylla, losing a few sailors eaten by Scylla rather than losing the entire ship swallowed up by the whirlpool of Charybdis. The ever-reasonable Erasmus (1469–1536) advised “The wise navigator will steer between Scylla and Charybdis…”. We aim to follow this injunction with AVRO. We judge the reluctance of adding four low-risk drugs to standard GB treatment to be the equivalent of sailing straight into Charybdis. We know the outcome of standard GB treatment in 2025.

Given (i) the benign nature of these four drugs, (ii) the current <2 year median survival of GB, (iii) the 84 studies showing that 24 different D2-blocking drugs inhibit GB growth and (iv) 10 studies showing GB growth inhibition by aprepitant, exploring the potential of AVRO drugs as adjuvants to standard GB treatment is reasonable. Although the AVRO drugs tend to be well tolerated, careful monitoring will be required.

With respect to D2, the 2023 paper of Yan Wang et al. [203] has great importance for both (i) understanding GB and (ii) understanding a puzzling finding in psychiatry. They outlined evidence for a dopaminergic, D2-centered amplifying feedback loop operating in GB. This loop is schematically depicted in Figure 3. They identified the cellular physiology of an amplifying feedback loop in GB in which dopamine signaling at D2 increased ERK pathway activation, which then resulted in increased dopamine production that furthers D2 signaling (Figure 3) [203]. This amplifying feedback cycle partly explains the characteristic GB D2 receptor overexpression [199,200,201,202,203,204,205].

With respect to psychiatry’s understanding of D2 in depression, Yan Wang et al. reported increased D2 expression in resected GB tissue from people with depression compared to GB tissue resected from people without depression. They further reported that the degree of D2 increase was inversely correlated with survival duration [203]. For several decades, standard psychiatric practice has included the addition of a D2 receptor-blocking antipsychotic drug in people with severe depression who have not, or only partially, responded to a first-line antidepressant drug. This addition frequently results in full resolution of treatment-resistant depression [316,317].

This demonstration of upregulated D2 expression in depression could explain this phenomenon. D2 upregulation is part of the depression-driven physiological changes that result in depression. D2 blockade reverses that driver. The feedback cycle of Wang et al. would also explain the well-known phenomenon in psychiatry that the longer an untreated psychosis persists, the more treatment-resistant it becomes.

The prevalence of depression in GB varies from 16% to 41%, and that of anxiety varies from 24% to 48% [318]. These comorbidities are major detriments to quality of life and, particularly for depression or anxiety, shorten overall survival [319]. Vortioxetine and olanzapine have good potential to improve these symptoms and even remain candidates for adding to the standard of care in GB on that basis.

Nausea after temozolomide, when it occurs, tends to be mild to moderate and is usually delayed to post-dose day 3, but when it occurs, it requires treatment. Nausea is more common and more severe in those with depression [320], making olanzapine plus vortioxetine of double benefit in those patients.

Seizures in GB patients occur in ~60% of patients [321]. Levetiracetam and lacosamide are preferred as first-line treatments because of their effectiveness, favorable pharmacokinetics, and lack of relevant interactions with chemotherapy drugs and dexamethasone or prednisone [322]. As there is a potential interaction between levetiracetam and vortioxetine/olanzapine, lacosamide may be a preferable seizure prophylaxis or treatment for patients under the AVRO regime.

Within a given patient’s GB reside an array of subpopulations. Each subpopulation has slightly different metabolic vulnerabilities, backup signaling pathways, and arrays of overexpressed drug efflux transporters [323,324]. Furthermore, GB cells have a stem cell shuttling ability—stem-to-nonstem and nonstem-to-stem.

In light of this, we ask, would not that wide set of GB attributes imply that, in effect, we face many different tumors within an individual case of GB? And would that not in turn imply the need for a multidrug approach such as adjunctive CUSP9v3 or AVRO? The conceptual foundation of the AVRO regimen lies in its multitarget approach to the complex biology of GB, integrating agents that reduce the action of key elements of tumor progression: inflammation, immune evasion, neurotransmitter dysregulation, and cellular proliferation. We reported data on the convergence of the effects of AVRO drugs on inhibiting tumor growth directly and reconditioning the GB niche toward reduced malignancy and increased therapeutic responsiveness.

The AVRO drug target dosages listed in Table 3 must be adjusted up or down to patient tolerability. Repurposing established drugs such as in AVRO for the treatment of cancer allows fast clinical translation, improved quality of life, and drug development cost benefits. These considerations plus the established safety of AVRO drugs justify a phase II study.

In sum, no predictable serious drug–drug interactions can be foreseen. Caution will be warranted when using other drugs that are metabolized by 3A4 in that both aprepitant and vortioxetine inhibit 3A4. Adding olanzapine and vortioxetine has good potential to improve quality of life in GB, independent from any anti-tumor effect.

## 7. Caveats

1. The 12 studies in Table 2 showing GB growth inhibition by the antipsychotic drug chlorpromazine prompted a 2023 clinical study by Pace et al. [325]. There was no clinical benefit from adding chlorpromazine 50 mg/day to GB’s standard of care [325]. Several problems limit the significance of this study. (i) As reviewed elsewhere, a multidrug regimen is needed to retard GB growth (in the absence of a silver bullet) [2,3,4,5,6,7,8,9]. (ii) The dose of chlorpromazine was too low to occupy and inhibit a significant percentage of the D2 receptors in the brain or tumor. (iii) As an inverse agonist at D1 [326], chlorpromazine would not be the ideal D2-blocking drug. Inverse agonism at D1 functionally lowers basal intracellular cAMP, the opposite of what we are aiming for.
2. The database for D2 blockade resulting in GB growth arrest or cell death includes studies ascribing this to autophagy inhibition, while other studies ascribe this to autophagy stimulation. How do we resolve this apparent contradiction?
3. Are we dealing with the metaphor of “blind men examining an elephant”? Are the many different MOAs ascribed to the inhibition of GB growth by antipsychotic drugs just the downstream consequences of a single D2 receptor event?
4. Some of the studies supporting GB inhibition by the AVRO drugs reported low microM range IC50 values. Ideally, we seek candidate drugs with low nM IC50 inhibition.
5. Most of the 84 studies shown in Table 2 and the 59 studies shown in Table 1 involved vitro cell culture or murine grafts. Often, such preclinical studies fail to translate into clinical benefit.

## 8. Conclusions

This paper has outlined a sound rationale for a small pilot study of the AVRO regimen as an adjunct to standard GB treatment. The data reviewed support the notion that the pharmacological maintenance of elevated intracellular cAMP represents a valuable goal throughout the disease course of GB and that both roflumilast and olanzapine contribute to achieving this goal. Furthermore, the evidence presented highlights the role of NK-1 signaling as a growth-promoting mechanism in GB. Each of the four drugs included in the AVRO regimen is well characterized in clinical practice, readily available, and associated with a low risk of adverse events. Taken together, these findings support the initiation of a pilot clinical study evaluating the safety and potential efficacy of adding AVRO to the current standard of care for GB.

## Figures and Tables

**Table 3:** Basic pharmacological parameters of AVRO drugs. All the values are approximate and the values vary widely from patient to patient. Listed doses are target doses. Most people will require dose reduction for one or more AVRO drugs.

Drug	Dose	T1/2	CYP Inhibition	CYP Metabolism
aprepitant	80 mg/d	~12 h	3A4	3A4
vortioxetine	20 mg/d	2–3 days	2D6	3A4, 2C19, 2D6
olanzapine	5 mg h/s.	~1 to 2 d	2D6	1A2
roflumilast	250–500 μg/d	~1 d	----	3A4, 1A2

## Data Availability

Data sharing is not applicable.

## Abbreviations

AVRO: the regimen of aprepitant, vortioxetine, roflumilast, olanzapine added to standard GB treatment; D2, dopamine receptor 2; GB, glioblastoma; HR, hazard ratio, rounded to tenth place; 5-HT, serotonin; MOA, mechanism of action; PDE, phosphodiesterase.
